# Recommendations for 46,XX Congenital Adrenal Hyperplasia Across Two Decades: Insights from the North American Differences of Sex Development Clinician Survey

**DOI:** 10.1007/s10508-024-02853-1

**Published:** 2024-04-29

**Authors:** Melissa Gardner, Behzad Sorouri Khorashad, Peter A. Lee, Barry A. Kogan, David E. Sandberg

**Affiliations:** 1 Department of Pediatrics, Susan B. Meister Child Health Evaluation and Research Center, University of Michigan Medical School, 2800 Plymouth Road, North Campus Research Complex, Building 16/G035E, Ann Arbor, MI 48109 USA; 2 Department of Pediatrics, Penn State Hershey Medical Center, Penn State College of Medicine, Hershey, PA USA; 3 Department of Urology, Albany Medical College and Center, Albany, NY USA; 4 Division of Pediatric Psychology, Department of Pediatrics, University of Michigan Medical School, Ann Arbor, MI USA

**Keywords:** Congenital adrenal hyperplasia, Disorders of sex development, Differences of sex development, Decision-making, Gender assignment, Clinical practice guidelines

## Abstract

Several aspects of clinical management of 46,XX congenital adrenal hyperplasia (CAH) remain unsettled and controversial. The North American Disorders/Differences of Sex Development (DSD) Clinician Survey investigated changes, over the last two decades, in clinical recommendations by specialists involved in the management of newborns with DSD. Members of the (Lawson Wilkins) Pediatric Endocrine Society and the Societies for Pediatric Urology participated in a web-based survey at three timepoints: 2003–2004 (T1, *n* = 432), 2010–2011 (T2, *n* = 441), and 2020 (T3, *n* = 272). Participants were presented with two clinical case scenarios—newborns with 46,XX CAH and either mild-to-moderate or severe genital masculinization—and asked for clinical recommendations. Across timepoints, most participants recommended rearing the newborn as a girl, that parents (in consultation with physicians) should make surgical decisions, performing early genitoplasty, and disclosing surgical history at younger ages. Several trends were identified: a small, but significant shift toward recommending a gender other than girl; recommending that adolescent patients serve as the genital surgery decision maker; performing genital surgery at later ages; and disclosing surgical details at younger ages. This is the first study assessing physician recommendations across two decades. Despite variability in the recommendations, most experts followed CAH clinical practice guidelines. The observation that some of the emerging trends do not align with expert opinion or empirical evidence should serve as both a cautionary note and a call for prospective studies examining patient outcomes associated with these changes.

**Supplementary Information:**

The online version contains supplementary material available at 10.1007/s10508-024-02853-1.

## Introduction

Congenital adrenal hyperplasia (CAH) is a group of autosomal recessive genetic disorders caused by a deficiency in one of five enzymes necessary for the synthesis of cortisol in the adrenal cortex. In over 95% of instances, CAH arises due to a deficiency in the enzyme 21-hydroxylase, causing a disruption in cortisol production. This disruption results in excess production of hormonal precursors, which subsequently convert into androgens. There are two forms of 21-hydroxylase CAH: the milder non-classic form, which is commonly diagnosed later in life and is often asymptomatic, and the classic form, which presents symptomatically soon after birth. The incidence of classic 21-hydroxylase CAH ranges between 1:14,000 and 1:18,000 and is subdivided into subtypes: the more severe salt-wasting and the milder simple-virilizing type. The ovaries and internal genital ducts of patients with 46,XX 21-hydroxylase CAH are largely unaffected, but increased prenatal androgens result in virilized genitalia at birth. This virilization may manifest as varying degrees of clitoromegaly, partially or completely fused labia, with rugation, and a confluence of the urethra and vagina (i.e., urogenital sinus) (Chan et al., 2020; Claahsen-van der Grinten et al., 2022).

These somatic features of CAH present multiple challenges for clinical teams and parents, especially in managing the psychosexual aspects of the condition, such as deciding the child’s gender of rearing, made particularly complex in cases involving highly virilized genitalia. Other difficult questions include when, if at all, genital surgical interventions should be performed, and at what age the child should be informed about any surgeries they may have experienced in infancy. The long-term adverse effects of surgical interventions, the issue of patient assent, and the ongoing debate over whether these surgeries are medically indicated or mainly intended to alleviate social discomfort contribute to these challenges (Sandberg & Vilain, 2022). Considering the relative rarity of this condition and the lack of systematic, standardized, longitudinal assessment of health outcomes among patients with CAH, firm evidence-based answers do not exist for most of these decisions (Speiser et al., 2018).

Disorders (or differences) of sex development (DSD) are defined as congenital conditions in which development of chromosomal, gonadal, or anatomic sex is atypical (Lee et al., 2006). Classic CAH—associated with genital virilization in females—is the most common cause of 46,XX DSD and is the most extensively studied DSD condition with regard to psychosocial and psychosexual outcomes. In addition to interest from physicians, individuals born with CAH have been the focus of study in multiple disciplines with implications for various fields of knowledge including psychology (Berenbaum & Beltz, 2011; Dessens et al., 2005; Hines et al., 2016), gender studies (Fausto-Sterling, 2015), feminism (Jordan-Young, 2012), elite sports (Karkazis et al., 2012), bioethics (Johnston, 2012), and gender politics (Lantos, 2013).

Regarding gender assignment, Speiser and White (2003) stated that most females with CAH ultimately identify as women, echoing the evidence cited in a 2002 statement by the Joint Workgroup of the Lawson Wilkins Pediatric Endocrine Society (North America) and the European Society for Paediatric Endocrinology that recommended rearing females with CAH as girls (Joint LWPES/ESPE CAH Working Group, 2002). In contrast, no specific gender of rearing was recommended in Endocrine Society clinical practice guidelines published in 2010 (Speiser et al., 2010) and 2018 (Speiser et al., 2018). Instead, consultation with a mental health provider with specialized expertise in DSD was recommended for psychosexual issues, including gender assignment at birth. Within the same month of publication of the 2010 Endocrine Society clinical practice guidelines, Houk and Lee (2010) proposed consideration of male gender assignment for 46,XX patients born with typical male-appearing external genitalia.

Regarding feminizing genital surgery and its timing (Table 1), the 2002 consensus statement (Joint LWPES/ESPE CAH Working Group, 2002) recommended that surgery be performed in infancy, between 2 and 6 months, and the clinical practice guidelines that followed in 2010 (Speiser et al., 2010) recommended genital surgery in infancy for cases with severe forms of genital virilization (i.e., Prader stage 3 or greater). The 2018 clinical practice guidelines (Speiser et al., 2018) made slightly different recommendations based on the degree of virilization: For those with mild virilization, the clinical practice guidelines advised informing parents about various surgical options including delaying the surgery until the child is older, and for those with severe virilization, it advised a discussion about early surgery to repair the urogenital sinus. Finally, the clinical practice guidelines “advise(d) that all surgical decisions remain the prerogative of families (i.e., parents and assent from older children) in joint decision-making with experienced surgical consultants” (Speiser et al., 2018). Worthy of note, all feminizing surgery recommendations in the 2018 clinical practice guidelines were labeled as an “Ungraded Good Practice Statement” (Guyatt et al., 2015).^1^

**Table 1:** Evolution of clinical practice guidelines for gender assignment and feminizing genital surgery in 46,XX CAH: 2002–2018

Years	Gender assignment	Surgery
2002–2003	“…most women are heterosexual, and their sexual identity is almost invariably female." (Speiser & White, 2003)^a^ “Even in females with psychosexual problems, general psychological adjustment seems to be similar to that of females without CAH. Currently, there is insufficient evidence to support rearing a 46,XX infant at Prader stage 5 as male.” (Joint LWPES/ESPE CAH Working Group, 2002)	“Early single-stage surgery between two and six months of life in girls with 21-hydroxylase deficiency, a time when the tissues are maximally pliable and psychological trauma to the child is minimized. Surgery during adolescence is often fraught with psychological and technical difficulties” (Speiser & White, 2003) “Based on recent clinical experience, the recommended time for surgery is at age 2–6 months; although, at present, this is not universal practice. It is important to note that surgery at this stage is technically easier than at later stages” (Joint LWPES/ESPE CAH Working Group, 2002)
2010	“We suggest that patients with CAH and psychosocial problems (such as gender assignment at birth) be referred to mental health staff with specialized expertise in managing such problems” (Speiser et al., 2010). ⨁⨁◯◯ “We propose consideration of male assignment for these 46,XX patients who have fully developed male genitalia based on available outcome data” (Houk & Lee, 2010)	“We suggest that for severely virilized (Prader stage 3) females, clitoral and perineal reconstruction be considered in infancy and performed by an experienced surgeon in a center with similarly experienced pediatric endocrinologists, mental health professionals, and social work services” (Speiser et al., 2010). ⨁⨁◯◯
2018	“For individuals with congenital adrenal hyperplasia and their parents, we recommend behavioral/mental health consultation and evaluation to address any concerns related to congenital adrenal hyperplasia” (Speiser et al., 2018). ⨁⨁◯◯	“In all pediatric patients with congenital adrenal hyperplasia, particularly minimally virilized girls, we advise that parents be informed about surgical options, including delaying surgery and/or observation until the child is older.” (*Ungraded Good Practice Statement*) “In severely virilized females, we advise discussion about early surgery to repair the urogenital sinus” (*Ungraded Good Practice Statement*) “In the treatment of minors with congenital adrenal hyperplasia, we advise that all surgical decisions remain the prerogative of families (i.e., parents Speiser et al. Guidelines on Congenital Adrenal Hyperplasia and assent from older children) in joint decision-making with experienced surgical consultants” (*Ungraded Good Practice Statement*)

^a^This 2003 paper (Speiser & White, 2003) is not a clinical practice guideline but a review describing CAH and its clinical management

The Grading of Recommendations Assessment, Development, and Evaluation (GRADE) directs authors of guidelines to assess the quality of evidence behind each recommendation (Guyatt et al., 2011): Recommendations are classified as very low (⨁◯◯◯), low (⨁⨁◯◯), moderate (⨁⨁⨁◯), and strong (⨁⨁⨁⨁)

Timing of disclosing early medical interventions to the child was not addressed in any of these CAH clinical practice guidelines. Although not a clinical practice guideline, per se, the 2006 “Consensus Statement on Management of Intersex Disorders” stated that “The process of disclosure concerning facts about karyotype, gonadal status, and prospects for future fertility is a collaborative, ongoing action that requires a flexible individual-based approach. It should be planned with the parents from the time of diagnosis” (Lee et al., 2006).

Growing cautiousness in the tone of clinical recommendations may reflect the inconclusive evidence supporting a specific approach. It may also reflect shifts in sociopolitical conceptualizations of sex and gender that have occurred in the last two decades. Notwithstanding substantial evidence suggesting that prenatal androgen exposure influences aspects of psychosexual differentiation in humans (Berenbaum & Beltz, 2021; Hines, 2020), it has been claimed that gender is neither an essential biologically determined quality nor an inherent identity; rather, it is repeatedly performed and reinforced by societal norms and sex is similarly culturally constructed (Morgenroth & Ryan, 2020). According to this doctrine, just as binary gender categories are maintained through social conditioning (via enforcing clear negative and stigmatizing consequences for failing to follow gender codes), binary sex categories are maintained by surgically reconstructing those who do not fit into the culturally constructed dichotomy of males and females (Butler, 2002; Morgenroth & Ryan, 2020). The example frequently used to support this notion is that the majority of newborns with “intersex traits” undergo surgery and are raised as either male or female, protecting and maintaining the binary construction of sex (Human Rights Watch, 2017). The United Nations (Méndez, 2013) and the Commissioner for Human Rights of the Council of Europe (Council of Europe & Commissioner for Human Rights, 2015) declared that the practices of early gonadal and genital surgery are human rights violations and forms of torture. These statements and their theoretical foundations have been challenged by the American Medical Association (American Medical Association, 2019) and several medical specialty organizations (North American Society for Pediatric Adolescent Gynecology, 2018; Societies for Pediatric Urology et al., 2017; Wolffenbuttel et al., 2018).

The aim of this study, which is part of a larger project, was to explore how medical and surgical experts whose specialties are central to the clinical management of CAH (i.e., pediatric endocrinology and urology) recommend managing various aspects of the clinical care of children born with 46,XX CAH, and how (if at all) these recommendations have changed over the last two decades. We investigated associations between participants’ medical specialty and their clinical recommendations and hypothesize that the physicians’ recommendations are based on the published clinical practice guidelines at the time of the study.

## Method

### Participants

Active members of the (Lawson Wilkins) Pediatric Endocrine Society and the Societies for Pediatric Urology, as listed in their respective membership directories, were targeted for participation at three timepoints: 2003–2004 (T1), 2010–2011 (T2), and 2020 (T3).^2^ Due to restrictions imposed by the endocrine society, recruitment for the 2020 wave of the survey was restricted to members who had previously been invited to participate in either 2003 or 2010. Inclusion criteria were (1) active membership of either society; (2) working within North America (US, Canada, Mexico); (3) having specialty training in endocrinology or urology; and (4) experience caring for patients with DSD. Unique survey links were emailed to a total of *n* = 706 (endocrinologists: 516; urologists: 190), *n* = 995 (endocrinologists: 777; urologists: 218), and *n* = 715 (endocrinologists: 434; urologists: 281) individuals in 2003, 2010 and 2020, respectively (Table 2 and Appendix, Participants [10.7302/21939]). The total number of eligible health providers who participated was *n* = 432 (endocrinologists: 300; urologists: 132) at T1; 441 (endocrinologists: 323; urologists: 118) at T2; and 272 (endocrinologists: 118; urologists: 154) at T3. Participation rates in T1, T2, and T3 were 58.1%, 41.6%, and 27.2% for endocrinology society members and 69.5%, 54.1% and 54.8% for urology society members. Of the total sample, 86 respondents participated at all timepoints.

**Table 2:** Recruitment and participation rates

	2003		2010		2020	
	PES	SPU	PES	SPU	PES	SPU
Named in directory	764	263	868	237	494^f^	354
Ineligible^a^						
Co-I, FG, or PT participant^b^	14	9	12	8	10	8
Emeriti	117	–	–	–	–	–
Retired	11	5	10	6	10	21
Deceased	2	–	1	–	–	–
No DSD patients	34	6	29	3	15	37
No clinical practice	16	1	16	–	4	3
Practice outside North America	1	–	13	–	8	2
Other: > 1 criteria met	53	52	10	2	13	2
Eligible sample	516	190	777	218	434	281
Participated	300 (58.1%)	132 (69.5%)	323 (41.6%)	118 (54.1%)	118 (27.2%)	154 (54.8%)
Logged in/consented only^c^	–	–	–	–	5	10
Declined participation^d^	48	10	27	3	5	5
No response	168	48	425	96	304	107
Eligible, but not invited^e^	0	0	1	1	0	0
No contact information	0	0	1	0	2	5

PES = Pediatric Endocrine Society; SPU = Societies for Pediatric Urology

^a^Ineligibility was determined at multiple stages. Determinations were made prior to sending survey invitations to some members; for others, it occurred after invitations were sent. It is possible that some of those for whom no responses were recorded were ineligible

^b^Co-investigators, focus group members, and pilot test participants involved in the design of this project

^c^At T3, several targeted participants had logged into the survey and completed portions of the screening survey or demographics but did not provide responses to items in any other section; this was not possible in earlier years

^d^A common reason cited for declining participation was being “too busy.”

^e^Reflects an error in recruitment

^f^Only members of PES who had been invited to participate in the past were included in the 2020 PES sample; this does not represent the total number of names listed in the 2020 PES directory

### Measures

#### Survey Development

Provisional survey items were generated based on a literature review and focus groups conducted by conference call. Focus groups were convened to identify themes pertinent to the investigation and canvass opinion regarding optimal survey administration format. Focus group participants included 16 junior and senior endocrine society and urology society members nominated for participation by colleagues who thought their opinions would be particularly informative; a geographically diverse sample was sought. Web-based administration to facilitate recruitment was the consensus of focus group participants. A preliminary survey was pilot tested with a subgroup of focus group participants with others checking for comprehensiveness of content coverage and survey response options. As such, focus group members were not eligible to participate in the actual survey. The final version of the survey comprised five sections (Table 3): (1) Demographics, (2) Clinical Case Presentations, (3) Factors Affecting Life Satisfaction, (4) Surgical Informed Consent, and (5) Mental Health Services and the DSD Team (for details on survey construction, see Appendix: Survey Development). Data from Sects. "Introduction" and "Method" are presented in this report.

**Table 3:** Clinician survey content overview

Section	Survey contents: major components		
Introduction	Overview of survey and eligibility screener^a^		
Demographics	Clinical practice and demographic characteristics		
Clinical case presentations	Cases:^b^	Mild-to-Moderate CAH	
		Severe CAH	
	Decisions:	Gender of rearing^c^	
		*In your professional judgment, which sex assignment/gender of rearing would result in the best long-term quality of life outcome [‘sex assignment’ does NOT necessarily imply genital surgery]?*	
		Surgical decision maker	
		*Who should decide whether genital surgery (hypospadias repair) should be performed?*	
		Timing of surgery (lists case-specific procedures)	
		*In your professional judgment, when should genital surgery be performed?*	
		Timing of disclosing surgical history to patient	
		*Genital surgery is sometimes completed at an early age such that the boy will have no memory of the procedure. If surgery had been performed at such an age in the case of this particular patient, do you think that information regarding the details of the surgery or karyotype should be disclosed to the patient? If so, when?*	
*Cases*^b^	Clinical case presentations		
Mild-to-Moderate CAH			Newborn with ambiguous genitalia identified by newborn screen and diagnosis of 21-hydroxylase congenital adrenal hyperplasia confirmed by Day 4. The clitoris was enlarged both in length (2 cm) and diameter, and there was partial posterior labioscrotal fusion
Severe CAH			The child, announced as a boy at birth, is first referred to you at 3 weeks of age with a salt-wasting crisis. The phallic structure measured 3.5 cm with fusion of the labial-urethral folds to the distal shaft (i.e., distal hypospadias). The labio-scrotal folds were fused and “scrotalized” so that complete scrotal development was present. Workup reveals 46,XX 21-hydroxylase congenital adrenal hyperplasia

CAH = Congenital Adrenal Hyperplasia

^a^Survey overview and instructions were included in all years; eligibility screen included at T3 (2020) only

^b^Labels “Mild-to-Moderate CAH” and “Severe CAH” are used here as shorthand to describe degree of genital virilization; these were not displayed in surveys materials seen by participants

^c^Gender of rearing recommendations were asked for the mild-to-moderate CAH case at T3 (2020) and for severe CAH in all years

#### Demographics

This section focused on participants’ personal (gender: male/female/other; birth year) and professional (number of DSD cases seen annually and over one’s entire career; specialty: endocrinology/urology; practice location: United States/Canada/Mexico/Other; and practice setting: solo or two-physician practice/group practice/HMO/medical school or hospital-based/other patient care employment/other non-patient care employment).

#### Case Presentations

This section comprised clinical scenarios, the first two of which involved 46,XX classic CAH: one with mild-to-moderate virilization and second with severe virilization (Table 3 and Appendix: Survey Items). The main variables assessed included recommended gender of rearing, surgical decision maker (parent or patient), genital surgery timing, and age at which to disclose to the patient their surgical history and karyotype (of those reared as boys).

Each case description was accompanied by color photos illustrating the degree of external genital virilization, followed by a series of multiple-choice questions asking for recommendations regarding: gender of rearing (boy/girl/other); who should decide whether genital surgery should be performed (parents in conjunction with physician/patient, likely during adolescence); timing of genital surgery (before 6 months/before 1 year/before school entry/during pre-adolescence: ages 6–10 years/adolescence: 11 years or older/I would recommend against surgery); and the age at which to disclose surgical details if surgery had been performed at so early age that the patient would not have a memory of the procedure, and the age at which to disclose karyotype to the patient (before school entry: 5 years/during middle childhood: 6–10 years/during adolescence: 11–17 years/during adulthood: 18 years or older/I would recommend against disclosure).

The survey was designed with automated conditional branching and skip patterns (Appendix: Fig. 2). Accordingly, responses to stem questions determined follow-up questions relevant to that choice alone: for example, those choosing the “girl” option in response to “which sex assignment/gender of rearing would result in the best long-term quality of life outcome?” would only be presented with questions on how to manage a girl with CAH, and those choosing the “patient” option in response to “who should decide whether genital surgery should be performed?” would not receive questions on timing of surgery, because including patients in the decision-making would necessitate a later timing of surgery. Although this branching and skip format more faithfully reflected actual clinical decision-making, it necessarily reduced the sample size for data analysis of particular items.

Across the three survey timepoints, limited changes were made to the case scenario response options for the gender of rearing question. For the mild-to-moderate CAH case, the gender of rearing question was not posed in either 2003 or 2010; questions regarding genital surgery presumed rearing the child as a girl. This question was asked in 2020 for the mild-to-moderate case and in all years for the severe case. In 2020, “other” was added as a response option alongside “girl” and “boy” for both cases. Those who chose “other” were not presented follow-up questions regarding genital surgery and disclosure.

### Procedure

Invitation letters that included an explanation of the study and survey login instructions were sent to Pediatric Endocrine Society and Societies for Pediatric Urology members in 2003–2004, 2010–2011, and 2020. Participants were also offered a paper-and-pencil version. To optimize recruitment, eligible respondents received up to three follow-up requests to participate. After survey completion rates declined to minimal levels over several weeks, we sent final requests to non-responders in the form of a concise, single-page letter. This letter aimed to encourage participation or prompt individuals to provide reasons for declining to participate (for details see Table 2).

### Data Analysis Plan

Participant demographic characteristics and responses to case scenario clinical management recommendations are summarized using descriptive statistics. Trends in recommendations and associations with the two main variables of interest (year of administration and provider specialty) and other participant characteristics (gender, age, clinical experience as measured by the number of cases per career, and practice setting) were examined using Generalized Estimating Equations (GEE). Accounting for the correlation between multiple responses from the same respondent (i.e., clustering by respondent), GEE have been recommended as a method for modeling longitudinal and categorical data (Agresti, 2002). In the case of the gender of rearing question, the “other” option was added in 2020: to accommodate this change, gender assignment is dichotomized as “girl” vs. “not girl.” Continuous data (e.g., physician age and number of cases seen over the career) were dichotomized using a median split to address outliers and categorized as “younger vs. older” (cut point: the birthyear 1952) and “less vs. more experienced” (cut point: 50 cases).

Options for the timing of surgery were classified into three categories: “early” (within the first year), “late” (after the first year), and a recommendation against surgery. The timing of disclosure options were similarly classified into three categories: “early” (before 18 years), “late” (after 18 years), and a recommendation against disclosure. Practice setting comprised “medical school or hospital-based” vs. “other.” For each item, the first model includes the predictor variables of survey timepoints, age, gender, specialty, practice setting, and clinical experience, and the second model includes the previous predictors in addition to the interaction between timepoints and specialty and timepoints and gender. All analyses were conducted using the Statistical Package for the Social Sciences (SPSS) for Windows software, Version 28.0.

## Results

### Demographic Characteristics of Participants

At all three time points, the majority of survey participants were male; this varied over time (Wald χ^2^(1) = 13.18, *p* = 0.001; male percentage: T1: 71.3%, T2: 61.7%, T3: 69.1%). At T1 and T2, more participants were endocrinologists; at T3, more were urologists (Wald χ^2^(1) = 70.93, *p* < 0.001; endocrinologist percentage T1: 69.4%, T2: 73.2% and T3: 43.4%) (see Participants for the explanation of reduced recruitment of Pediatric Endocrine Society members in 2020). The majority reported their practice setting as medical school or hospital-based with no significant change over time (Wald χ^2^(1) = 5.35, *p* = 0.069; T1: 67.1%, T2: 73.5%, and T3: 77.9%). The mean age of participants was higher at T3 (T1: 51.9 years; T2: 51.1; T3: 56.3). A significant interaction between time and specialty was found (Wald χ^2^(2) = 64.22, *p* < 0.001) for age, where urologists (T1: 51.5; T2: 54.3; T3: 54.1) were older at T2, but younger at T3 compared to endocrinologists (T1: 52.1; T2: 50.0; T3: 59.3). Over time, there also was a statistically significant shift in the clinical experience of the participants, indexed by the number of patients cared for throughout their career: T2 respondents reported less experience compared to those in the T1 and T3 surveys (T1: 50.0; T2: 42.5; T3: 50.0; Wald χ^2^(1) = 9.62, *p* = 0.008) (Table 4).

**Table 4:** Participant demographic and professional characteristics at each survey timepoint

		2003 (*n* = 432)		2010 (*n* = 441)		2020 (*n* = 272)	
		*n*	%	*n*	%	*n*	%
Specialty	PES	300	69.4	323	73.2	118	43.4
	SPU	132	30.6	118	26.8	154	56.6
Gender	Male	308	71.3	272	61.7	188	69.1
	Female	124	28.7	169	38.3	84	30.5
	Other^a^	–	–	–	–	1	0.4
Practice setting	Medical school or hospital	290	67.1	324	73.5	212	77.9
	Other	142	32.9	117	26.5	60	22.1
Practice country	United States	407	94.2	417	94.6	256	94.1
	Canada	25	5.8	24	5.4	16	5.9
		Mean	SD	Mean	SD	Mean	SD
Birth year		1951	9	1959	10	1964	10
Clinical experience (DSD cases/career)		50	100	43	167	50	337

PES = Pediatric Endocrine Society; SPU = Societies for Pediatric Urology; DSD = Differences/disorders of sex development; SD = Standard deviation

^a^Other” was added as a response option in 2020

### Gender of Rearing Recommendations: Boy vs Girl vs Other (Fig. 1)

**Fig. 1:**
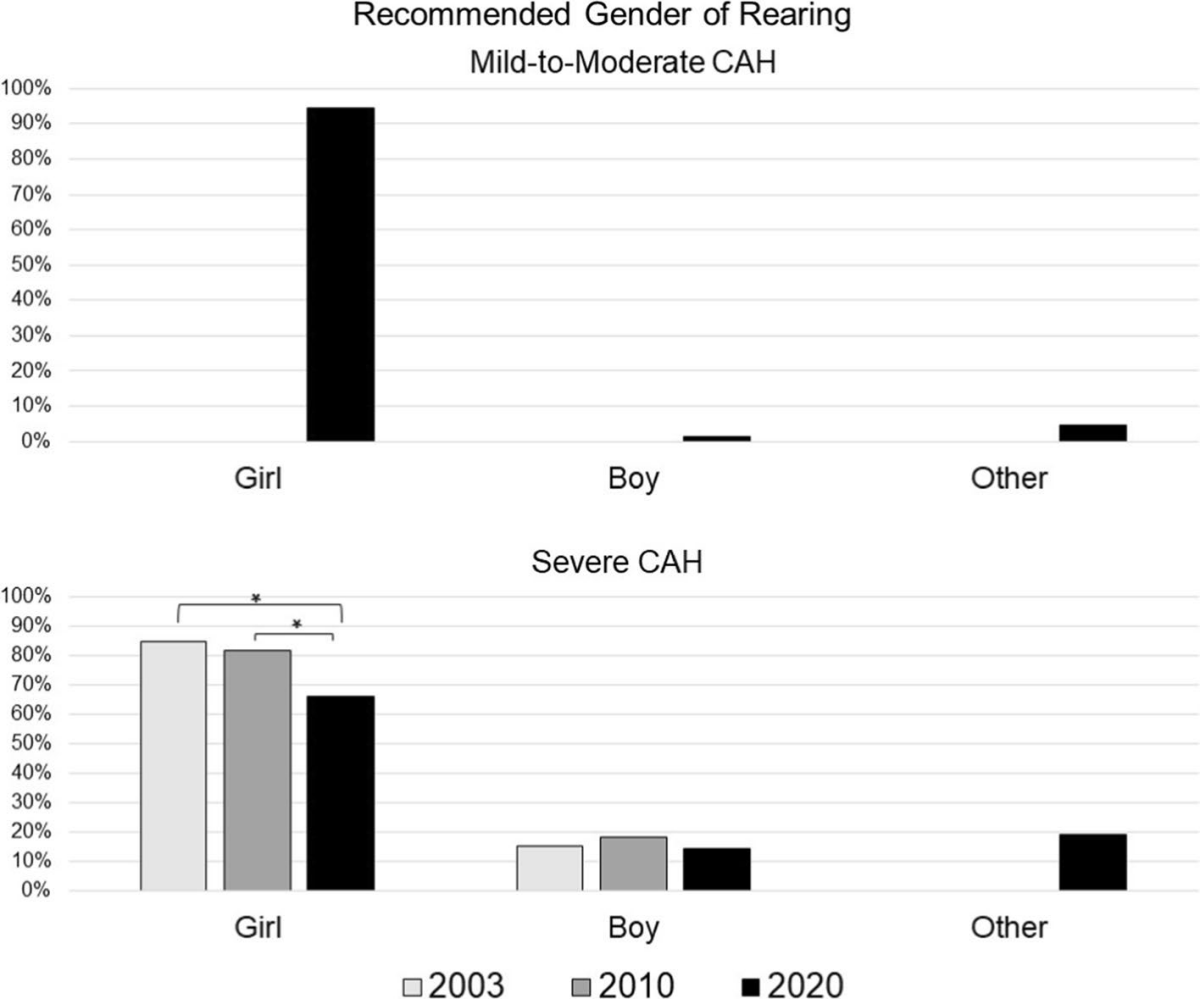
Recommended gender of rearing for CAH with mild-to-moderate and severe virilization. Gender of rearing was assessed for mild-to-moderate CAH only in 2020; “Other” was added as an option for both cases only in 2020. * Statistically significant between-group differences, *p* < .001

#### Mild-to-Moderate CAH

Recommended gender assignment for this case was only asked in 2020. The majority (94%) recommended rearing the child as a “girl,” 1% as a “boy,” and 4% as “Other (e.g., Intersex, non-binary).” None of the predictor variables were statistically significant.

#### Severe CAH

Across study timepoints, most providers recommended rearing the child as a girl (T1: 85%; T2: 82%; T3: 66%); however, a statistically significant shift over time was evident in recommending a gender other than girl (Wald’s χ^2^(2) = 30.98, *p* < 0.001). No other predictor was statistically significant (Table 5).

**Table 5:** Likelihood of recommending rearing each case as a girl across survey timepoints and participant demographic characteristics

Comparison		Mild-to-Moderate CAH				Severe CAH			
		OR^a^	Lower	Upper	*p*	OR	Lower	Upper	*p*
Year of survey administration	2003–2020	NA^b^				2.755	1.863	4.075	< .001
	2010–2020					2.128	1.54	2.941	< .001
	2010–2003					1.295	0.906	1.85	NS
Specialty	PES to SPU	0.855	0.178	4.106	NS	1.185	0.798	1.758	NS
Gender	Male to Female	1.039	0.212	5.091	NS	1.236	0.827	1.847	NS
Age^c^	Young to Old	0.449	0.096	2.109	NS	1.008	0.653	1.556	NS
Experience^d^	Less to More	1.125	0.395	3.201	NS	0.888	0.649	1.216	NS
Practice setting	Medical School or Hospital to Other	2.295	0.713	7.391	NS	0.774	0.534	1.123	NS

CAH = Congenital Adrenal Hyperplasia, OR = Odds ratio; PES = Pediatric Endocrine Society; SPU = Societies for Pediatric Urology

^a^The likelihood of recommending rearing each case as a girl, in the first category (e.g., PES) compared to the second category (e.g., SPU)

^b^Recommended gender of rearing was only asked in 2020 for the mild-to-moderate scenario; it was asked in all years for the severe scenario

^c^Median split was used to categorize participants into the younger and older age groups

^d^Median split of cases seen over participant’s career was used to categorize respondents into the lesser and more experienced groups

### Surgical Decision-Making: Parents or Patients (Fig. 2)

#### Mild-to-Moderate CAH

*Recommending rearing as girl* Although most participants, at all three timepoints, recommended parents are responsible for decision-making (T1: 87.5%; T2: 79%; T3: 67%), a statistically significant trend toward involving the patient was evident (Wald’s χ^2^(2) = 50.79, *p* < 0.001). At all three timepoints, urologists (T1: 96.2%; T2: 89.8%; T3: 79.9%) were more likely than endocrinologists (T1: 83.7%; T2: 74.9%; T3: 50%) to recommend that parents make surgical decisions (in consultation with physicians) rather than deferring to the child when older (Wald’s χ^2^(1) = 24.11, *p* < 0.001) (Table 6) .

**Table 6:** Likelihood of recommending the patient lead surgical decision-making across survey timepoints and participant demographic characteristics

Comparison		Mild-to-moderate CAH				Severe CAH							
		Reared as girl				Reared as girl				Reared as boy			
		OR^a^	Lower	Upper	*p*	OR	Lower	Upper	*p*	OR	Lower	Upper	*p*
Year of survey administration	2003 to 2020	0.247	0.167	0.367	< 0.001	0.199	0.105	0.375	< 0.001	0.474	0.219	1.026	NS
	2010 to 2020	0.382	0.27	0.54	< 0.001	0.472	0.28	0.795	0.005	0.539	0.253	1.147	NS
	2010 to 2003	1.545	1.106	2.158	0.011	2.376	1.362	4.145	0.002	0.88	0.433	1.787	NS
Specialty	PES to SPU	3.059	1.958	4.78	< 0.001	1.515	0.776	2.959	NS	2.676	1.162	6.163	0.021
Gender	Male to Female	0.737	0.505	1.076	NS	0.649	0.37	1.139	NS	0.905	0.409	2.001	NS
Age^b^	Young to Old	1.582	1.007	2.484	0.047	2.023	0.963	4.25	NS	3.297	1.248	8.709	0.016
Experience^c^	Less to More	1.271	0.934	1.729	NS	1.068	0.689	1.653	NS	0.777	0.396	1.524	NS
Practice Setting	Medical School or Hospital to Other	0.911	0.631	1.316	NS	1.431	0.838	2.445	NS	1.136	0.516	2.499	NS

CAH = Congenital Adrenal Hyperplasia, OR = Odds ratio; NS = not significant; PES = Pediatric Endocrine Society; SPU = Societies for Pediatric Urology

^a^The likelihood of recommending the patient lead surgical decision-making in the first category (e.g., PES) compared to the second category (e.g., SPU)

^b^Median split was used to categorize participants into the younger and older age groups

^c^Median split of cases seen over one’s career was used to categorize participants into the lesser and more experienced groups

**Fig. 2:**
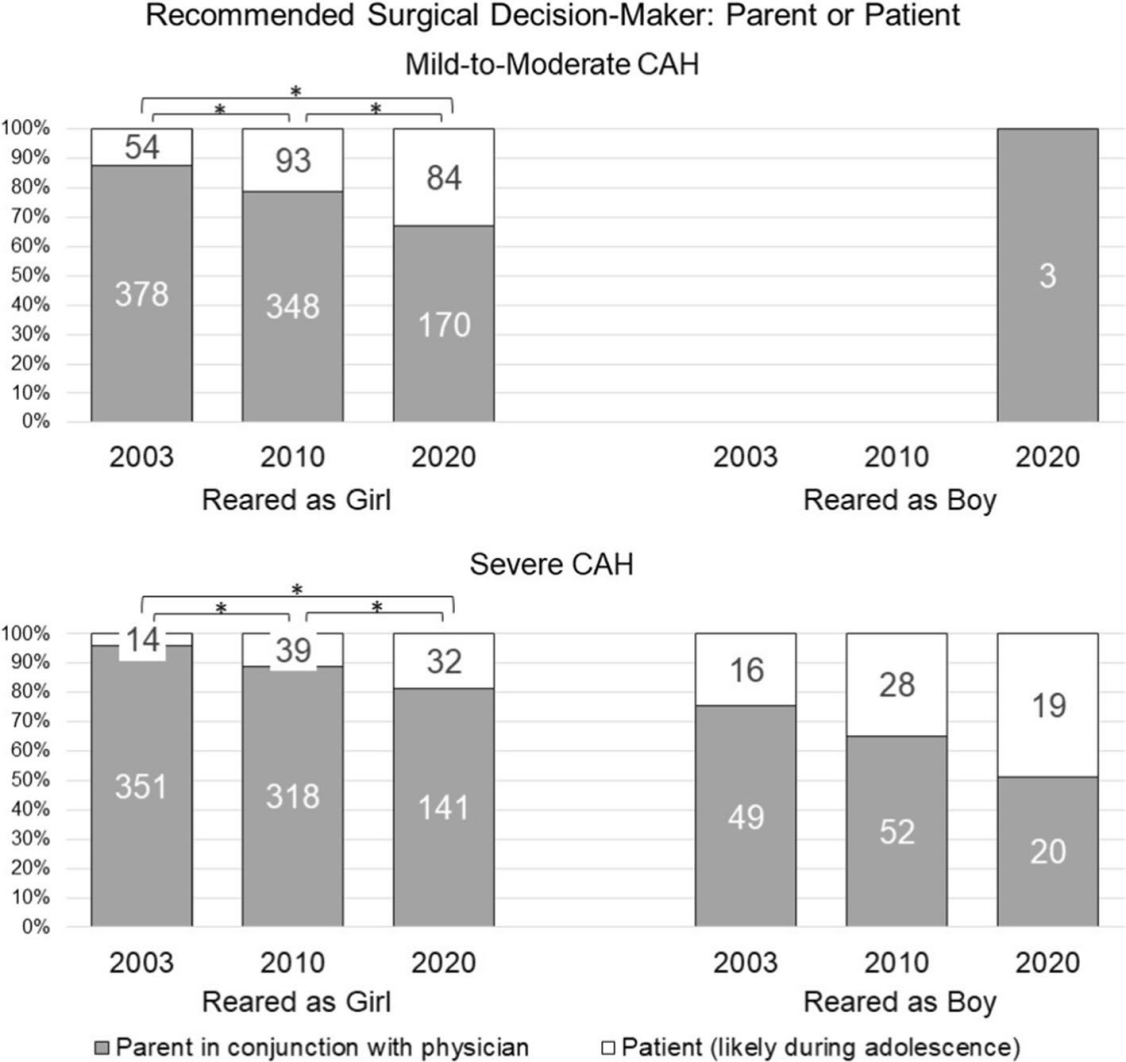
Recommendations regarding surgical decision maker. For mild-to-moderate CAH, gender of rearing was presumed to be “girl” in 2003 and 2010; participant recommendations were assessed in 2020; for severe CAH, gender of rearing was assessed for all participants. * Statistically significant between-group differences, *p* < .001

*Recommending rearing as boy* Only 3 participants recommended this option: all recommended that parents in conjunction with physician specialists should make decisions.

#### Severe CAH

*Recommending rearing as girl* Most recommended that parents should make decisions at all timepoints (T1: 96%; T2: 89%; T3: 82%), but an increase in likelihood of including the patient in the decision-making process was also evident (Wald’s χ^2^(2) = 24.90, *p* < 0.001). No other significant effects were detected.

*Recommending rearing as boy* Most recommended that parents should make decisions about genital surgeries at each timepoint without any statistically significant change over time (T1: 75%; T2: 65%; T3: 51%). Compared to endocrinologists (T1: 68.9%; T2: 65.5%; T3: 31.8%), urologists (T1: 90%; T2: 63.6%; T3: 76.5%) were more likely to prioritize parents in decision-making (Wald’s χ^2^(1) = 5.35, *p* = 0.021).

### Surgical Timing Recommendations: Before or After One Year of Age (Fig. 3)

**Fig. 3:**
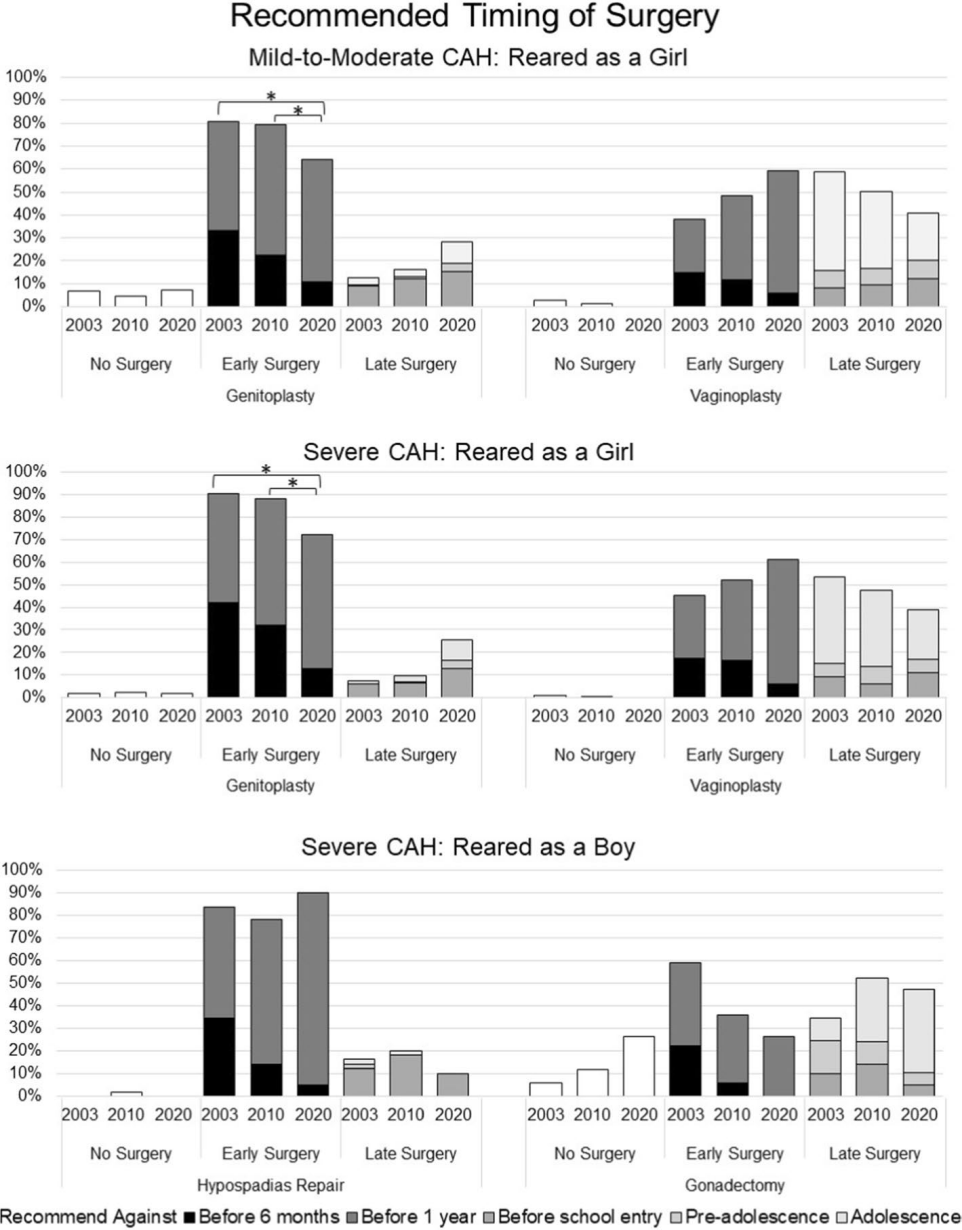
Recommendations regarding timing of surgeries. * Statistically significant between-group differences, *p* < .001

#### Mild-to-Moderate CAH

*Recommending rearing as girl* At each timepoint, most recommended performing early genitoplasty/clitoroplasty (T1: 81%; T2: 79%; T3: 64%), although, a statistically significant decline was observed between T2 and T3 (Wald’s χ^2^(2) = 13.45, *p* = 0.001). No other significant effects were detected. In contrast, when vaginoplasty was the surgical focus, an increasing percentage recommended early surgery (T1: 38%; T2: 49%; T3: 59%), although this change was not statistically significant. Urologists at each timepoint (T1: 61.4%; T2: 64.8%; T3: 65.5%) were more likely to recommend early vaginoplasty than endocrinologists (T1: 26.5%; T2: 41.5%; T3: 46.2%; Wald’s χ^2^(1) = 17.21, *p* < 0.001).

*Recommending rearing as boy* Among the three participants who recommended rearing this case as a boy, one recommended against any surgery and two recommended surgeries before one year.

#### Severe CAH

*Recommending rearing as girl* Most recommended early genitoplasty/clitoroplasty (T1: 90.6%; T2: 88.3%; T3: 72.1%); however, a statistically significant decline for this recommendation occurred over time (Wald’s χ^2^(2) = 18.65, *p* < 0.001). No other predictors yielded statistically significant effects. In the case of vaginoplasty, early surgery trended toward being more frequently recommended over time (T1: 45.30%; T2: 52.10%; T3: 61.30%), although the increase was not statistically significant. Urologists (T1: 60.2%; T2: 65.5%; T3: 64.0%) were more likely than endocrinologists (T1: 38.6%; T2: 47.2%; T3: 56.9%) to recommend early vaginoplasty (Wald’s χ^2^(1) = 6.52, *p* = 0.011). Similarly, male respondents (T1: 47.8%; T2: 57.1%; T3: 61.5%) were more likely than female respondents to recommend early vaginoplasty (T1: 38.3%; T2: 43.7%; T3: 60.6%; Wald’s χ^2^(1) = 4.51, *p* = 0.034; Wald’s χ^2^(1) = 6.63, *p* = 0.01).

*Recommending rearing as boy* The majority recommended early hypospadias repair surgery with no statistically significant change over time (T1: 84%; T2: 78%; T3: 90%). Male respondents (T1: 88.2%; T2: 82.8%; T3: 93.3%) were more likely than female respondents to recommend early surgery (T1: 73.3%; T2: 71.4%; T3: 80%) at each timepoint (Wald’s χ^2^(1) = 6.06, *p* = 0.014). No other significant differences were detected.

Regarding removal of the ovaries (i.e., gonadectomy), there was a decrease in the proportion of those recommending early gonadectomy (T1: 59%; T2: 36%; T3: 26%), but this apparent trend was not statistically significant. Male respondents (T1: 61.8%; T2: 42.9%; T3: 35.7%) were more likely than females to recommend early gonadectomy (T1: 53.3%; T2: 27.3%; T3: 0%) (Wald’s χ^2^(1) = 4.23, *p* = 0.04). No other significant effects were found.

### Disclosing Early Surgery and Discordant Karyotype (Fig. 4)

**Fig. 4:**
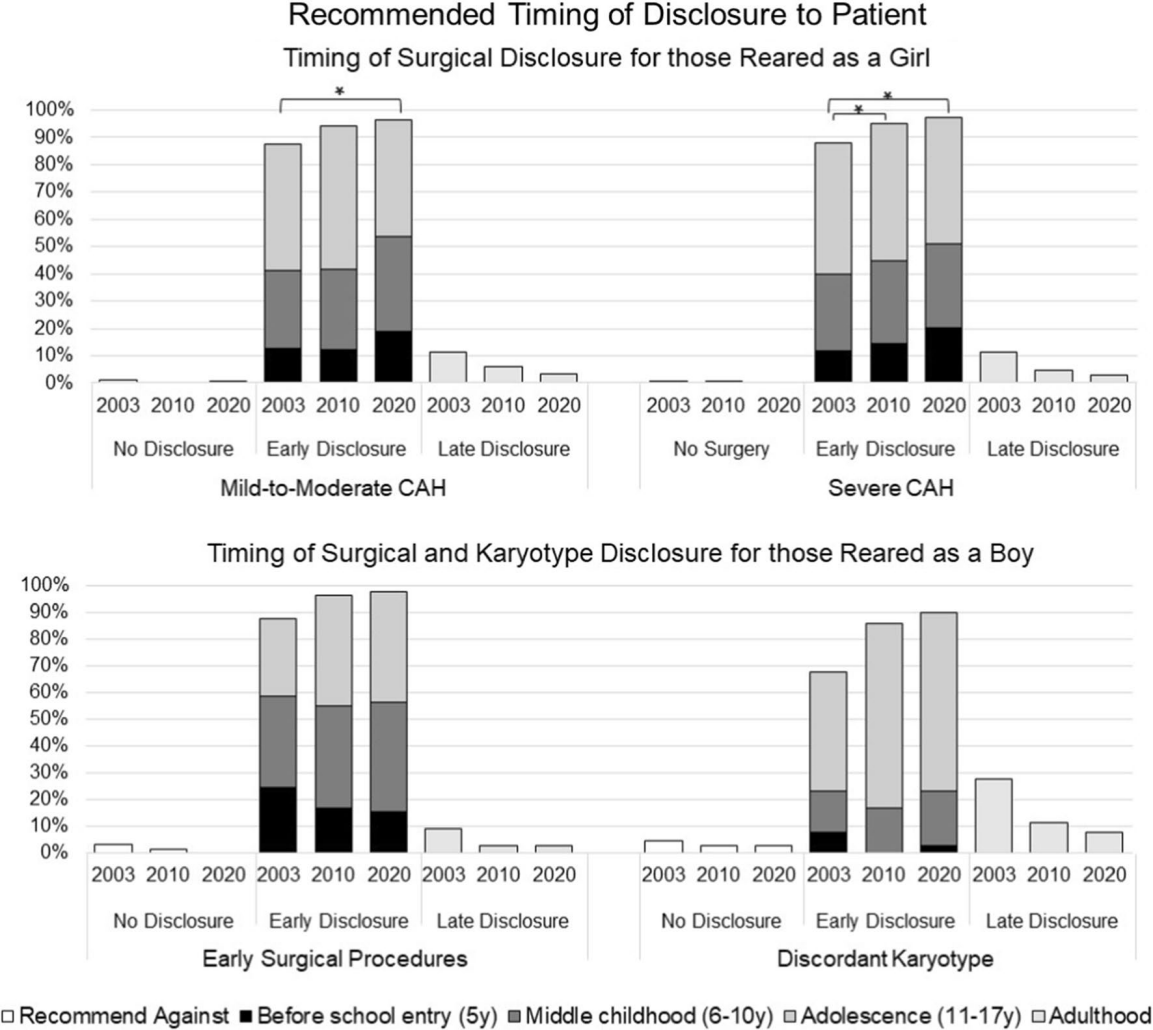
Recommended timing for disclosure of surgical history and karyotype. * Statistically significant between-group differences, *p* < .05

#### Mild-to-Moderate CAH

*Recommended rearing as girl* At each timepoint, most respondents recommended disclosure (regarding early surgery) before the patient reaches 18 years old (T1: 88%; T2: 94%; T3: 96%) with a significant increase over time (Wald’s χ^2^(2) = 6.41, *p* = 0.041). No other statistically significant effects were detected.

*Recommending rearing as boy* All three participants recommending male gender assignment also recommended disclosing the karyotype and history of early surgical procedures to the patient before 18 years of age.

#### Severe CAH

*Recommended rearing as girl* At each timepoint most respondents recommended early disclosure of surgeries (T1: 88%; T2: 95%; T3: 97%) with a statistically significant increase across survey waves (Wald’s χ^2^(2) = 16.84, *p* < 0.001). No other significant effects were found.

*Recommending rearing as boy* At each timepoint, most respondents recommended early disclosure (before 18 years of age) of surgeries (T1: 88%; T2: 96%; T3: 97%) and the patient’s chromosomal sex (T1: 68%; T2: 86%; T3: 90%). No other statistically significant effects were detected.

### Subsample Participating in all Three Surveys

A total of 86 participants participated in all three surveys. The median birth year of these participants was 1957; 55.8% were endocrinologists, and 75.6% identified as men. In brief, there was a decline in recommending a female gender assignment for severe CAH (T1: 84.9%; T2: 75%; T3: 68.2%; Wald’s χ^2^(2) = 6.93, *p* = 0.031); an increase in involving the patient in decision-making for both CAH cases when the recommendation was to rear as a girl (mild-to-moderate: T1: 15.1%; T2: 18.6%; T3: 32.5%, Wald’s χ^2^(2) = 13.10, *p* = 0.001; severe: T1: 4.1%; T2: 11.1%; T3: 15.8%; ns); a decrease in recommending early surgery for both cases when rearing as a girl was recommended (mild-to-moderate: T1: 89%; T2: 82.6%; T3: 62.3%, Wald’s χ^2^(2) = 6.66, *p* = 0.036; severe: T1: 92.9%; T2: 92.9%; T3: 68.8%, Wald’s χ^2^(2) = 10.81, *p* = 0.004); and an increase in recommending early disclosure for both cases reared as girls (mild-to-moderate: T1: 94.2%; T2: 92.9%; T3: 95.1%, ns; severe: T1: 94.5%; T2: 93.7%; T3: 96.5%, ns) (See Supplementary Table 1 and 2, 10.7302/21939).

## Discussion

Over the course of two decades, this survey assessed the clinical recommendations of North American pediatric endocrinologists and urologists. The focus was on two cases of 46,XX CAH: one with mild-to-moderate virilization and the other with severe virilization of the external genitalia. For the severely virilized case, there was a statistically significant increase over time among those recommending a gender other than “girl.” Regarding genital surgery, the majority at all three timepoints, and for both the mild-to-moderate and severe cases, recommended that the parents arrive at a decision (in conjunction with the physician) and that early surgery is preferred; yet an increase over time was detected in the proportion of participants recommending that the patient be the one to decide on surgery rather than the parents and physicians and this was associated with a significant increase in the proportion recommending that surgery occur at later ages. In addition to changes over time, urologists, at each timepoint, were more likely to recommend parents serve as decision makers, and an earlier age for vaginoplasty, compared to endocrinologists. These results indicate that while most participants’ recommendations align with clinical practice guidelines, emerging trends, such as assigning a gender other than “girl” and postponing surgical interventions to allow the patient to decide, deviate from or contest these guidelines.

*Gender assignment* A statistically significant increase in recommending a gender other than girl was observed across survey timepoints. According to several review articles, the percentage of individuals with CAH, assigned female at birth, who go on to experience gender dysphoria can vary from 5 to 10% (Almasri et al., 2018; Babu & Shah, 2021; Dessens et al., 2005). The reason for this shift away from recommending the gender “girl” is unclear. Moreover, at the T3, when the option “other” was added to “male” and “female,” 5% of respondents recommended rearing the child with mild/moderate virilization as “other (e.g., Intersex, non-binary),” and 20% recommended this option for the severely virilized case. To the best of our knowledge, no published studies exist that focus on the long-term psychological adjustment of individuals raised as genders other than "boy" or "girl." Since the option to choose "other" for gender of rearing was only introduced in the T3 survey, it is speculative to say whether participants would have made similar recommendations in earlier years.

These survey findings may also be particularly surprising given historical perspectives on the subject. For instance, a leading intersex advocate stated in 2003 that “all children should be assigned as male or female, without surgery”(Chase, 2005) (p. 240). Furthermore, the 2006 Consensus Statement on Management of Intersex (Lee et al., 2006), which included representatives from the intersex advocacy community in both the U.S. and Europe, did not mention the option of raising a child with a DSD as anything other than male or female.

*Surgical decision-making* The majority of survey participants recommended that parents should be the primary authority for surgical decision-making. Nevertheless, a statistically significant trend was observed in recommending that this authority be transferred to the patient, when older. Also, endocrinologists were more likely than urologists, to recommend including patients in the decision-making. It is crucial to note that involving patients in decision-making inevitably leads to a preference for postponing early surgery. Therefore, the respondents' choice of parents over patients as the authority for decision-making may be related to the asserted benefits of early surgical outcomes (Elsayed et al., 2020; Rink, 2011). An increase in recommending that the patient be the final decision maker was also observed within the smaller sample of participants who participated in all three surveys.

White papers on pediatric decision making published by the Council on Ethical and Judicial Affairs of the American Medical Association support the recommendation of the majority of our respondents regarding the authority of parents in making decisions on behalf of their young children (American Medical Association, 2007, 2019). The Endocrine Society’s most recent CAH clinical practice guidelines (Speiser et al., 2018) similarly recommended that, “In the treatment of minors with congenital adrenal hyperplasia, we advise that all surgical decisions remain the prerogative of families (i.e., parents and assent from older children) in joint decision-making with experienced surgical consultants” (Recommendation 7.3). This recommendation was categorized as an “Ungraded Good Practice Statement” (Guyatt et al., 2015). A consequence of the current lack of RCTs and the unlikelihood that RCTs could ever occur in this area is that the field will need to move forward based on expert consensus, clinical experience, and/or accepted best practice.

*Surgical timing* According to the Endocrine Society’s most recent CAH clinical practice guidelines (Speiser et al., 2018), “In all pediatric patients with congenital adrenal hyperplasia, particularly minimally virilized girls, we advise that parents be informed about surgical options, including delaying surgery and/or observation until the child is older. (Ungraded Good Practice Statement)” (Recommendation 7.1). Although in our sample, most recommended early genitoplasty for girls with CAH, a shift toward postponing genitoplasty/clitoroplasty was evident: in the mild-to-moderate case, 127 out of 432 (29.4%) in T1, 165 out of 441 (37.4%) in T2, and 143 out of 254 (56.3%) in T3 recommended either late surgery (including those recommending the patient as the decision maker) or were opposed to any surgery. In the severe case, out of those who had recommended a girl gender of rearing, 47 out of the 365 (13%) in T1, 76 out of 357 (21%) in T2, and 71 out of 178 (40%) in T3 recommended either late surgery (including those recommending the patient as the decision maker) or were opposed to any surgery. In contrast, recommending early vaginoplasty did not significantly change across time, for either of the cases. This observation comports with the 2018 recommendation “In severely virilized females, we advise discussion about early surgery to repair the urogenital sinus. (Ungraded Good Practice Statement)” (Recommendation 7.2).

A recent analysis of surveys of patients with DSD (including 46,XX CAH), concerning views on the timing of genital surgery, indicates that prohibiting genital surgery at a young age may not align with the preferences of a significant number of these patients (Meyer-Bahlburg, 2022). For instance, a survey of 14 DSD-specialized clinics in six European countries reported that 46% of the 173 participants with CAH believed that surgeries should be performed in infancy (before 6 months of age) and an additional 20% believed surgeries should be performed in childhood. Less than 10% stated that the surgeries should be done in “adulthood” (5%) or “at any age the patient decides” (4%) (Bennecke et al., 2021). Emphasizing the preference for early surgery even further, 74% of 151 female participants with CAH who had received surgeries, disagreed with the sentence, “I think I would have been better off without any of the surgeries performed in my childhood/adolescence,” and 51% of them disagreed with the sentence, “Any decision about surgical procedures should be postponed until the affected person reaches the age of legal responsibility” (Bennecke et al., 2021). The contrast between emerging trends among clinicians and the findings from studies of adults who had received early genital surgery suggests that the shift in recommendations may be influenced by the dissemination of speculative messages in various media and efforts at legislating bans on early elective genital surgery (e.g., proposed California legislation [Equality California, 2021, January 15]). Even if adults who had received such procedures express a preference for the procedures being performed before they could provide assent or legal consent, it may be understandable that clinicians are less likely to recommend an option which has been equated by the United Nations to “torture.” (Council of Europe & Commissioner for Human Rights, 2015; Human Rights Watch, 2017; Méndez, 2013).

Although our findings are based on surveys and hypothetical clinical scenarios, they align with some emerging evidence suggesting a trend toward postponing genitoplasty in real-world clinical settings: A chart review study at a single Midwestern tertiary care medical center found that, between 1979 and 2013, there was a linear decline in the rate of clitoroplasty in CAH patients which the authors attribute to the “power of patient advocacy” (Schoer et al., 2018).

### Disclosure

An area of tension voiced by intersex advocates concerns failures to fully share information with the patient. From the earliest stages of the intersex advocacy movement, “honest, complete disclosure” was recommended as a strategy to prevent the patient from experiencing their medical condition as shameful (Chase, 2003). Because genital surgery is often completed at an early age such that children may have little memory of procedures, survey respondents were given the opportunity to recommend the age when information about genital surgery (genitoplasty/hypospadias repair or vaginoplasty) should be shared with the patient. In our study, regardless of condition severity and gender assignment, most recommended early disclosure at all three timepoints. Moreover, a significant increase in favor of earlier disclosure was detected for both cases such that by 2020, this recommendation was almost universal. In addition to the calls from intersex activists, empirical evidence supports the value of early disclosure: Data from 903 individuals with DSD obtained from 14 DSD clinics in Europe demonstrated that openness about the condition is associated with better mental health and lower anxiety and depression (van de Grift, 2023).

### Limitations

The findings of this study need to be considered within the context of its limitations. One potential limitation to consider is the participation rates. At T1, 58% and 69% of the pediatric endocrinologists and urologists, respectively, participated. These proportions fell to 42% and 54% at T2 and 27% and 56% in T3. The relatively high percentage of non-responders may suggest the risk of non-response bias. Notwithstanding the substantially lower participation rate among endocrinologists in the T3, the proportion of eligible participants completing our surveys is actually higher than studies also targeting members of both the Pediatric Endocrine Society (Marks et al., 2019; Singer et al., 2019) and Societies for Pediatric Urology (Haslam et al., 2021). Very few studies have reported higher participation rates (e.g., Diamond et al., 2006). A meta-analysis of surveys has shown that the mean participation rate in those using email is 33% and in those using traditional mail is 53% (Shih & Fan, 2009), and according to a systematic review of response rates in patient and healthcare professional surveys in surgery, the average response in 1,746 surveys on clinicians was 53% (Meyer et al., 2022). The limitation on endocrinologist recruitment at T3 resulted in the addition of no new endocrinologists being recruited in 2020; in contrast, new urologist members were recruited and included. Associated with this restriction, the endocrinologist sample is both older and more experienced than in previous years and as compared with the urologist sample at T3 (see Appendix, Participant Demographics). It is possible that this may have affected statistical results and their interpretation. The results from our sub-sample analysis, focusing on those who participated in all three surveys, align with the trends observed in the larger sample. This suggests that the changes in recommendations cannot solely be attributed to different generations of physicians but also to individual conceptualizations of the condition.

As in any other survey study, there are also concerns that responses to scenario-based clinician surveys do not accurately reflect “real-world” decision making. A defense of this methodology goes beyond the scope of this report; however, as noted above, there is emerging evidence that trends observed in our survey are mirrored in studies suggesting similar changes in ongoing care. More generally, studies of clinician judgments and decision making using scenarios, such as those in the present study, have been shown to be generalizable (Evans et al., 2015).

### Conclusion

Despite variability in the recommendations, the majority of expert responses follow CAH clinical practice guidelines. However, there are growing trends for some recommendations which are at odds with these. There is a slowly growing trend to recommend rearing a child with severe 46,XX CAH in a gender other than boy or girl, as well as to perform genital surgery later in life. Given that evidence or expert opinion is lacking regarding the wisdom of these shifting recommendations, it remains to be seen whether these trends are evident in real life clinical management and, if so, whether they will result in better outcomes for patients compared with current standards of care.

### Supplementary Information

Below is the link to the electronic supplementary material.

Supplementary file1 (PDF 429 kb)

Supplementary file2 (DOCX 21 kb)

## Data Availability

Code is available upon request.

## Footnotes

1 In the context of clinical practice guidelines, a "Good Practice Statement" that is labeled as "Ungraded" is usually a recommendation for which there is not sufficient evidence to assign a formal grade based on standard evidence-ranking systems. Clinical practice guidelines often rank the strength of their recommendations based on the quality and quantity of evidence available to support those recommendations. Various systems such as the GRADE (Grading of Recommendations Assessment, Development and Evaluation) system (Guyatt et al., 2011) are used to evaluate this evidence. Recommendations can be graded based on a number of criteria, including the methodology of the studies they are based on, the consistency of study results, and the directness of the evidence. However, there are some situations where a strong clinical recommendation needs to be made even though there is insufficient evidence to grade the recommendation in a standard way. These are often basic care recommendations that are so fundamental to good care that they do not require randomized controlled trials or other high-level evidence to support them. In such cases, the guideline committee might issue a "Good Practice Statement" without a grade, acknowledging that the recommendation is based on expert consensus, clinical experience, and/or accepted best practice rather than on graded evidence. The lack of a grade should not be interpreted as meaning that the recommendation is not important or not valid; rather, it signifies that the recommendation is considered to be a fundamental aspect of good patient care for which formal grading is not applicable.

2 The Lawson Wilkins Pediatric Endocrine Society was founded in 1972; in 2010 the name of the Society was changed to the Pediatric Endocrine Society (PES).

## Data Availability

Data are available upon request.
