# Supplementary material for: Recommendations for 46,XX Congenital Adrenal Hyperplasia Across Two Decades: Insights from the North American Differences of Sex Development Clinician Survey
Source: Arch Sex Behav. 2024 Apr 29;53(5):1695–711. doi: 10.1007/s10508-024-02853-1 (PMC11106198; doi:10.1007/s10508-024-02853-1)
Supplement: Supplementary file 2 — Supplementary file2 (DOCX 21 kb) [file 10508_2024_2853_MOESM2_ESM.docx]

Supplementary Table 1. Likelihood of recommending rearing each case as a girl across different timepoints and participant demographic characteristics

| Comparison | | Mild-to-Moderate CAH | | | |  | Severe CAH | | | |
| --- | --- | --- | --- | --- | --- | --- | --- | --- | --- | --- |
|  |  | **OR^1^** | **Lower** | **Upper** | ***p*** |  | **OR** | **Lower** | **Upper** | ***p*** |
| Year of survey administration | 2003 to 2020 | NA^2^ | | | |  | 2.755 | 1.863 | 4.075 | <.001 |
|  | 2010 to 2020 |  |  |  |  |  | 2.128 | 1.54 | 2.941 | <.001 |
|  | 2010 to 2003 |  |  |  |  |  | 1.295 | 0.906 | 1.85 | NS |
| Specialty | PES to SPU | 0.855 | 0.178 | 4.106 | NS |  | 1.185 | 0.798 | 1.758 | NS |
| Gender | Male to Female | 1.039 | 0.212 | 5.091 | NS |  | 1.236 | 0.827 | 1.847 | NS |
| Age^3^ | Young to Old | 0.449 | 0.096 | 2.109 | NS |  | 1.008 | 0.653 | 1.556 | NS |
| Experience^4^ | Less to More | 1.125 | 0.395 | 3.201 | NS |  | 0.888 | 0.649 | 1.216 | NS |
| Practice setting | Medical School or Hospital to Other | 2.295 | 0.713 | 7.391 | NS |  | 0.774 | 0.534 | 1.123 | NS |
| *A subsample of participants who attended all three timepoints (n=86)* | | | | | | | | | | |
| Year of survey administration^a^ | 2003 to 2020 |  |  |  |  |  | 2.589 | 1.274 | 5.261 | 0.009 |
|  | 2010 to 2020 |  |  |  |  |  | 1.437 | 0.834 | 2.478 | 0.192 |
|  | 2010 to 2003 |  |  |  |  |  | 0.555 | 0.282 | 1.092 | 0.088 |
| Specialty | PES to SPU | 0.314 | 0.023 | 4.211 | NS |  | 1.116 | 0.475 | 2.623 | NS |
| Gender | Male to Female | All females recommended “girl”^5^ | | | |  | 1.21 | 0.481 | 3.045 | NS |
| Age^3^ | Young to Old | 1.405 | 0.107 | 18.519 | NS |  | 1.932 | 0.717 | 5.203 | NS |
| Experience^4^ | Less to More | All those with less experience recommended “girl”^5^ | | | |  | 0.908 | 0.518 | 1.589 | NS |
| Practice setting | Medical School or Hospital to Other | 0.489 | 0.036 | 6.727 | NS |  | 0.75 | 0.304 | 1.847 | NS |

Abbreviations: CAH = Congenital Adrenal Hyperplasia, OR = Odds ratio; PES = Pediatric Endocrine Society; SPU = Societies for Pediatric Urology

^1^ The likelihood of recommending rearing each case as a girl, in the first category (e.g., PES) compared to the second category (e.g., SPU)

^2^ Recommended gender of rearing was only asked for the mild-to-moderate CAH case in 2020; it was asked for the severe CAH case in all years.

^3^ Median split was used to categorize participants into the younger and older age groups

^4^ Median split of cases seen over respondent’s career was used to categorize participants into the less and more experienced groups

^5^ In these cases, all members of one category had chosen one answer

Supplementary Table 2. Likelihood of recommending the patient lead surgical decision-making across different timepoints and participant demographic characteristics

| Comparison | | Mild-to-Moderate CAH | | | |  | Severe CAH | | | | | | | | |
| --- | --- | --- | --- | --- | --- | --- | --- | --- | --- | --- | --- | --- | --- | --- | --- |
|  |  | **Reared as girl** | | | |  | **Reared as girl** | | | |  | **Reared as boy** | | | |
|  |  | **OR^1^** | **Lower** | **Upper** | ***p*** |  | **OR** | **Lower** | **Upper** | ***p*** |  | **OR** | **Lower** | **Upper** | ***p*** |
| Year of survey administration | 2003 to 2020 | 0.247 | 0.167 | 0.367 | <.001 |  | 0.199 | 0.105 | 0.375 | <.001 |  | 0.474 | 0.219 | 1.026 | NS |
|  | 2010 to 2020 | 0.382 | 0.27 | 0.54 | <.001 |  | 0.472 | 0.28 | 0.795 | 0.005 |  | 0.539 | 0.253 | 1.147 | NS |
|  | 2010 to 2003 | 1.545 | 1.106 | 2.158 | 0.011 |  | 2.376 | 1.362 | 4.145 | 0.002 |  | 0.88 | 0.433 | 1.787 | NS |
| Specialty | PES to SPU | 3.059 | 1.958 | 4.78 | <.001 |  | 1.515 | 0.776 | 2.959 | NS |  | 2.676 | 1.162 | 6.163 | 0.021 |
| Gender | Male to Female | 0.737 | 0.505 | 1.076 | NS |  | 0.649 | 0.37 | 1.139 | NS |  | 0.905 | 0.409 | 2.001 | NS |
| Age^2^ | Young to Old | 1.582 | 1.007 | 2.484 | 0.047 |  | 2.023 | 0.963 | 4.25 | NS |  | 3.297 | 1.248 | 8.709 | 0.016 |
| Experience^3^ | Less to More | 1.271 | 0.934 | 1.729 | NS |  | 1.068 | 0.689 | 1.653 | NS |  | 0.777 | 0.396 | 1.524 | NS |
| Practice Setting | Medical School or Hospital to Other | 0.911 | 0.631 | 1.316 | NS |  | 1.431 | 0.838 | 2.445 | NS |  | 1.136 | 0.516 | 2.499 | NS |
| *A subsample of participants who attended all three timepoints (n=86)* | | | | | | | | | | | | | | | |
| Year of survey administration | 2003 to 2020 | 0.313 | 0.158 | 0.621 | <.001 |  | 0.215 | 0.055 | 0.831 | 0.026 |  | 0.81 | 0.11 | 5.75 | NS |
|  | 2010 to 2020 | 0.43 | 0.247 | 0.749 | 0.003 |  | 0.645 | 0.22 | 1.894 | NS |  | 0.39 | 0.09 | 1.78 | NS |
|  | 2010 to 2003 |  |  |  |  |  | 2.54 | 0.862 | 5.156 | NS |  |  |  |  | NS |
| Specialty | PES to SPU | 1.523 | 11.343 |  | 0.005 |  | 1.033 | 0.274 | 3.9 | NS |  | 7.10 | 0.81 | 62.37 | NS |
| Gender | Male to Female | 0.684 | 0.257 | 1.823 | NS |  | 0.28 | 0.083 | 0.94 | 0.039 |  | 3.44 | 0.46 | 25.73 | NS |
| Age^2^ | Young to Old | 1.1 | 0.366 | 3.309 | 0.039 |  | All “old” participants chose “parent” | | | |  | 4.68 | 0.32 | 68.30 | NS |
| Experience^3^ | Less to More | 1.414 | 0.79 | 2.531 | NS |  | 1.345 | 0.597 | 3.026 | 0.475 |  | 0.31 | 0.07 | 1.45 | NS |
| Practice Setting | Medical School or Hospital to Other | 0.396 | 0.164 | 0.954 | NS |  | 1.017 | 0.217 | 4.765 | 0.983 |  | 1.79 | 0.23 | 14.07 | NS |

Abbreviations: CAH = Congenital Adrenal Hyperplasia, OR = Odds ratio; NS = Not Significant; PES = Pediatric Endocrine Society; SPU = Societies for Pediatric Urology

^1^ The likelihood of recommending the patient lead surgical decision-making in the first category (e.g., PES) compared to the second category (e.g., SPU)

^2^ Median split was used to categorize participants into the younger and older age groups

^3^ Median split of cases seen over respondent’s career was used to categorize participants into the less and more experienced groups
